# Pathogenicity and Virulence of *Legionella*: Intracellular replication and host response

**DOI:** 10.1080/21505594.2021.1903199

**Published:** 2021-04-12

**Authors:** Deepika Chauhan, Stephanie R. Shames

**Affiliations:** Division of Biology, Kansas State University, Manhattan, Kansas, USA

**Keywords:** Legionella, pathogenicity, virulence, host-response, innate immunity

## Abstract

Bacteria of the genus *Legionella* are natural pathogens of amoebae that can cause a severe pneumonia in humans called Legionnaires’ Disease. Human disease results from inhalation of *Legionella*-contaminated aerosols and subsequent bacterial replication within alveolar macrophages. *Legionella* pathogenicity in humans has resulted from extensive co-evolution with diverse genera of amoebae. To replicate intracellularly, *Legionella* generates a replication-permissive compartment called the *Legionella*-containing vacuole (LCV) through the concerted action of hundreds of Dot/Icm-translocated effector proteins. In this review, we present a collective overview of *Legionella* pathogenicity including infection mechanisms, secretion systems, and translocated effector function. We also discuss innate and adaptive immune responses to *L. pneumophila*, the implications of *Legionella* genome diversity and future avenues for the field.

## Introduction

Bacteria of the genus *Legionella* are natural pathogens of environmentally free-living eukaryotes that can cause respiratory illness in humans termed legionellosis. Legionellosis includes a fatal pneumonia called Legionnaires’ Disease (LD) and a self-limiting illness called Pontiac Fever. LD primarily affects elderly and immunocompromised individuals, including those on immunosuppressive therapy[1]. *Legionella* bacteria were first isolated from an outbreak of atypical pneumonia at the 1976 American Legion Convention in Philadelphia, USA[2]. The etiological Gram-negative bacillus was identified and named *Legionella pneumophila* after the disease and its victims[3]. LD accounts for 2–9% of total community-acquired pneumonia; however, the worldwide prevalence of LD is difficult to quantify due to underdiagnosis, variation in diagnostic methods, awareness level, and reporting standards between countries[4]. It is predicted that less than 5% of cases are properly reported and diagnosed [1,5]. Pontiac Fever is a benign, febrile, non-pneumonic disease caused by exposure to *Legionella* bacteria, which is rarely diagnosed and does not require antimicrobial treatment[2]. *Legionella* carry a massive toolbox of virulence factors that facilitate its survival and robust intracellular replication [6,7]. This review provides an overarching discussion of *Legionella* pathogenicity and the interactions of these pathogens with the mammalian immune system.

Human disease is primarily a consequence of *Legionella* colonization of anthropomorphic freshwater environments, including air-conditioning cooling towers, building water systems and spa pools [8,9]. *Legionella* infection occurs almost exclusively from aspiration of contaminated water and person-to-person transmission is very rare[10]. The rarity of transmission between humans and co-evolution with unicellular amoebae has likely resulted in *Legionella*’s susceptibility to innate immune defenses (see below). Consequently, *Legionella* spp. are clinically important pathogens that additionally serve as valuable models to dissect mechanisms of both host–pathogen interactions and innate immune defense.

Approximately half of the 65 identified *Legionella* species have been associated with human disease; however, the overwhelming majority of clinical infections (~90%) are caused by a single species, *L. pneumophila*. The next most common etiological agents of LD are *L. longbeachae, L. bozemanii* and *L. micdadei*, which account for 2–7% of infections worldwide[11]. Interestingly, *L. longbeachae* is the leading cause of LD (~30%) in Australia and New Zealand and is the only species naturally found in soil [12,13]. In comparison to *L. pneumophila*, pneumonia caused by non-*pneumophila Legionella* (non-*Lpn*) species are rare and almost exclusively nosocomial[11]. Interestingly, *L. pneumophila* and non-*Lpn* species are found in similar habitats, including built freshwater environments; however, non-*Lpn* species contribute less to overall disease burden. This can be attributed to various factors such as difficulty in strain recovery from water samples, decreased fitness of some species in the sediment of aquatic systems, lack of serological test validation of non-*Lpn* spp., and high genetic diversity among *Legionella* species, which makes diagnosis difficult [11,14].

*Legionella* spp. are ubiquitous in the environment where they parasitize and replicate within free-living eukaryotic phagotrophs, primarily Amoebozoa [15,16]. Amoebae including *Acanthamoeba castellanii, Acanthamoeba polyphaga, Hartmannella vermiformis, Dictyostelium discoideum, and Naegleria* spp. are natural hosts of *Legionella* and are used to investigate *Legionella* host–pathogen interactions[17]. Amoebae serve a dual role for *Legionella* by providing both a niche for intracellular replication and protection from harsh conditions of the environment including antibiotics, chemicals, heat, and osmotic stress [18–21]. *Legionella*’s environmental persistence is also due to colonization of interspecies biofilms in natural and built freshwater environments [22,23]. Within biofilms *Legionella* are phenotypically heterogeneous, containing subpopulations of virulent non-growing bacteria[24]. The phenotypic variation in biofilms is controlled by *Legionella* quorum sensing (Lqs) system along with a transcription factor, LvbR, and the temperature[24]. The nongrowing cells or “persisters” are metabolically active, have high tolerance to antibiotics and express virulence genes. These virulent sessile persisters are highly infectious and replicate efficiently within permissive protozoan hosts[24]. *Legionella*’s extensive adaptation to persist and replicate within natural freshwater environments has resulted in efficient colonization of built environments and consequent human disease.

All sequenced *Legionella* species encode a highly conserved type IVB secretion system (T4SS) called Dot/Icm (defective for organelle trafficking/intracellular multiplication) [25,26]. The Dot/Icm T4SS is essential for intracellular replication, spans both bacterial membranes and functions to translocate hundreds of bacterial virulence factors, termed effector proteins, directly into host cells. Dot/Icm-translocated effectors have diverse functions and biochemical activities but broadly act to subvert lysosomal bacterial degradation and acquire nutrients from the host cell (see below). *L. pneumophila* encodes over 300 individual effector genes, which comprise 10% of open reading frames in the genome. Despite conservation of the Dot/Icm T4SS and relative abundance of effector genes, there is extensive interspecies variation in translocated effector repertoires[26]. *Legionella* spp. encode the greatest quantity and diversity of effectors of all intracellular pathogens characterized to date [25,26].

## Intracellular lifecycle of *Legionella*

### Attachment to host cells

**Figure 1.**
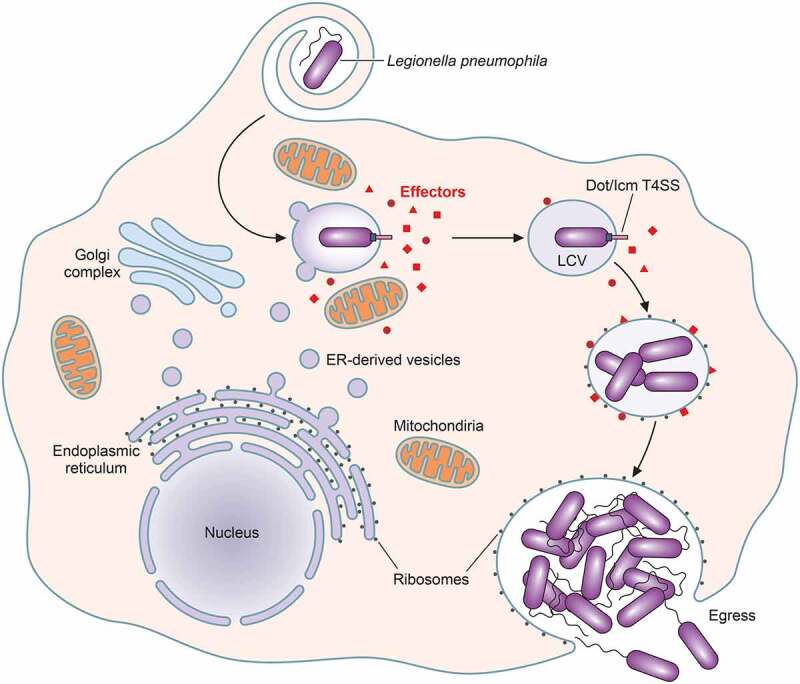
**The life cycle of *Legionella pneumophila* within eukaryotic host cells**. Bacterial uptake takes place by either coiling (shown) or conventional phagocytosis. Early after entry into the host cell, *L. pneumophila* loses its flagella and the *Legionella* containing vacuole (LCV) escapes the endocytic pathway via effector-mediated recruitment of endoplasmic reticulum (ER)-derived vesicles and transient association with mitochondria. Subsequently, the LCV becomes studded with ribosomes and effectors and exponential replication occurs (replicative phase; see text). Upon exhaustion of host nutrients, *L. pneumophila* become flagellated (transmissive phase; see text) and egress the host cell

The basic mechanism of *L. pneumophila* intracellular replication is consistent between natural (amoebae) and accidental hosts (mammalian macrophages). Following initial attachment and phagocytosis, *Legionella* intracellular replication is contingent on biogenesis and maintenance of its replicative niche, the *Legionella* containing vacuole (LCV), and temporal regulation of egress (Figure 1)[27].

Initial attachment of *L. pneumophila* to host cells is enhanced by several bacterial factors. The *rtxA locus, pilEL locus, ladC*, and *enhC*, which encode a type I secretion system, type IV pili, an inner membrane-associated protein and a periplasmic protein, respectively, facilitate *L. pneumophila* adherence and entry into both amoebae and mammalian cells [28–31]. *L. pneumophila* major outer membrane protein (MOMP), a porin, and Lcl, a collagen-like protein contribute to mammalian cell adhesion [32–34]. However, *L. pneumophila* also exploits host factors for its attachment and internalization, including the complement receptors CR1 and CR3, which are engaged by MOMP, and Fc receptors. Complement and Fc receptors are important for *L. pneumophila* entry into macrophages since antibody-mediated neutralization of CR1, CR3 or Fc receptors impairs phagocytosis [35–38]. Opsonin-independent adherence to macrophages and lectin-mediated adherence to *A. castellanii* has been described; however, specific factors involved in this process have yet to be definitively characterized [37,39–42]. Together, initial attachment of *L. pneumophila* to host cells is facilitated by both host and bacterial factors.

### *Phagocytosis of* Legionella

After attachment, *L. pneumophila* enters the host cell by either traditional phagocytosis or a specialized process called coiling phagocytosis [43,44]. In contrast to traditional phagocytosis, a symmetrical and circumferential uptake processes, coiling phagocytosis involves encircling of extracellular bacteria by unilateral pseudopods making this as an asymmetrical engulfment process (Figure 1)[45]. However, the biological relevance of coiling phagocytosis is unclear since it is neither necessary for intracellular replication nor a pathogen-driven process as heat-killed bacteria are also internalized by this mechanism [20,43,46].

Phagocytosis of *L. pneumophila* is an active process relying on phosphatidylinositol 3-kinase (PI3K)-mediated actin polymerization. Chemical inhibition of either PI3K (LY294002 or wortmannin) or actin polymerization (cytochalasin-D) impair *L. pneumophila* phagocytosis [39,47–49]. Actin binding proteins called coronins are also important for *L. pneumophila* phagocytosis. Coronins are transiently recruited to the phagocytic cup of U937 macrophage-like cells harboring live, but not dead, *L. pneumophila*, which supports a role for directed atypical phagocytosis in *L. pneumophila* pathogenicity[50]. Furthermore, the Dot/Icm T4SS is important for efficient *L. pneumophila* uptake by phagocytes and at least one translocated effector (SdeA/LaiA) contributes to this process[51]. Thus, *L. pneumophila* phagocytosis is a directed uptake process and is conserved at the molecular level among diverse host cells.

### LCV biogenesis: Evasion of endocytic trafficking and vacuole acidification

*L. pneumophila* utilizes a myriad of virulence mechanisms to escape the endocytic pathway and establish the LCV. Canonically, phagocytosed bacteria are delivered to early endosomes where the early sorting process occurs. While some receptors are recycled back to the surface, the remaining cargo is transported to late endosomes and lysosomes for degradation [52]. Thus, phagosomes rapidly undergo endocytic maturation and conversion to a phagolysosome. The acidic microenvironment of phagolysosomes along with lysosomal hydrolases efficiently degrade internalized particles, including bacteria[53]. However, the LCV evades endocytic maturation through effector-mediated subversion of vesicular trafficking, ribosomal localization, the ubiquitin-proteasome system, phosphoinositide metabolism and vacuolar acidification.

Rapid molecular remodeling of *Legionella*-containing phagosomes is essential for LCV biogenesis and bacterial intracellular replication (Figure 1)[54–56] *L. pneumophila* temporally regulates the pH of the LCV. Despite the importance of lysosomal evasion and maintenance of near neutral vacuolar pH at early stages of infection [<6 h post-infection (p.i.)] [55,56], vacuolar acidification at late time points is important at late stages. Concomitantly, at late time points during infection (>18 h p.i.), LCVs acidify and acquire late endosomal and lysosomal markers such as lysosomal-associated membrane protein 1 (LAMP-1)[56]. Treatment of infected macrophages with bafilomycin, a vacuolar(v)-ATPase inhibitor, which prevents acidification, impairs bacterial replication[56]. Dot/Icm-translocated effectors also temporally regulate LCV pH through subversion of the v-ATPase. The effector SidK is expressed at early stages of infection and prevents acidification through binding v-ATPase VatA, which impairs v-ATPase function[57]. WipB, a lysosome-targeted phosphatase, also interacts with components of the host v-ATPase but the influence of these interactions on v-ATPase function are unknown[58]. This suggest that regulation of v-ATPase activity is a key feature for intracellular replication of *L. pneumophila* in macrophages.

Initial studies imaging fixed *L. pneumophila-*infected cells revealed Dot/Icm-mediated recruitment of at least one mitochondrion to the LCV [59,60]. However, recent live-cell imaging revealed that mitochondrial association with the LCV is transient, highly dynamic and independent of the Dot/Icm T4SS [59,61–63]. Dot/Icm-dependent subversion of mitochondrial metabolism at early time points contributes to *L. pneumophila* intracellular replication. The effector LegG1 induces dynamin 1-like protein (DNM1L)-dependent mitochondrial fragmentation. The abrupt halt in mitochondrial respiration leads to a Warburg-like metabolism in macrophages, which favors bacterial replication [61,64].

Multiple *L. pneumophila* effectors modulate vesicular and organelle trafficking [65–69]. Many effectors that subvert host vesicular trafficking have been identified through yeast secretion assays and localization to either the Golgi apparatus or ER [66,68,70–72]. While the molecular mechanisms by which these effectors function have not been fully elucidated, recent advances have revealed a critical role for host phosphoinositide metabolism (reviewed in [73]), small GTPases (see below and reviewed in [74,75]) and the retromer complex [76,77].

Subsequently, ER-derived vesicles are replaced by ribosomes and the LCV transitions into a replication-permissive a rough-ER-like compartment [59,78]. Recruitment of ribosomes to the LCV is a conserved process in both amoeba and macrophages; however, the mechanism by which it occurs is still not clear [79,80]. *Legionella* effectors likely play a role in this process as *dot*/*icm* mutants do not establish an LCV [59,62]. Moreover, establishment of the LCV is conserved between *Legionella* species. Despite genomic differences, [81] the *L. longbeachae* LCV is similar to the *L. pneumophila* LCV as it avoids lysosomal fusion and Rab1 and Sec22b are recruited [81,82]. These findings highlight the conservation of essential pathways and phenotypic similarities among *Legionella* species.

*L. pneumophila* effectors regulate the function of several host small GTPases to facilitate LCV biogenesis [75,83]. Modulation of small GTPases is central to LCV biogenesis and several comprehensive reviews have been published on this topic [74,75,84,85]. Immediately following *L. pneumophila* entry, Rab1 and Arf1 are recruited to the LCV [54,86]. In macrophages, recruitment of Rab1 and Arf1 to LCVs is mediated by the effectors SidM/DrrA (defect in Rab1 recruitment) and RalF, respectively. LidA binds to the cytoplasmic face of the LCV and synergizes with SidM to recruit Rab1 [87,88]. The effector, VipD, has Rab5-dependent phospholipase A1 activity that inhibits endosomal fusion of LCV by catalyzing removal of PI(3)P from the endosomal membrane[89]. Rab5 and Rab7 play an important role in phagosome maturation and are required for cargo transition from early to late endosomes. These small GTPases also play a role in retrograde trafficking of endosomes to the Golgi-complex [90,91].

Effector-mediated subversion of the host retromer complex is important for LCV biogenesis. The effector RidL impedes retrograde trafficking pathway by binding to the retromer subunit Vps29 and the lipid phosphoinositol-3-phosphate [PI(3)P], which localizes retromer components to the LCV membrane [76,92]. This, in turn, modulates retromer function and promotes intracellular replication of *L. pneumophila*, likely through LCV acquisition of retrograde transport vesicles [77,93].

### *Biphasic lifecycle of* Legionella

*Legionella* have a biphasic lifecycle that alternates between an infectious transmissive phase and a non-infective replicative phase. In nutrient-rich conditions, such as within host cells, *Legionella* undergoes exponential replication (replicative phase) and under scarcity of nutrients the bacteria enter into stationary (transmissive) phase. After exit from the nutrient deprived host, *Legionella* disperse into the environment and reestablish infection into a new host cell, which offers a protective intracellular niche favorable for replication[94].

The transition between growth phases is accompanied by major transcriptomic changes. Nearly half of *L. pneumophila*’s predicted genes have a drastic shift in expression from replicative to transmissive phase[95]. In the replicative phase, genes related to metabolism, amino acid degradation/breakdown, sugar assimilation, cell division and biosynthetic processes are upregulated. During transmissive phase, genes associated with host entry, virulence and survival, which include Dot/Icm-translocated effectors, motility machinery (flagellar and type IV pilus genes), enhanced entry proteins (Enh) and cyclic-di-GMP regulatory proteins are upregulated[95].

*Legionella’s* transition from replicative to transmissive phase is a highly coordinated process that is initiated upon nutrient limitation. Amino acid starvation triggers synthesis and accumulation of guanosine 3,5-bispyrophosphate (ppGpp), which initiates stationary phase and consequent up-regulation of virulence genes[96]. Similar to other microbes, *L. pneumophila* ppGpp synthetases, RelA and SpoT, are activated when uncharged tRNAs bind to ribosomes[97]. Moreover, transition from replicative to transmissive phase is controlled by the sigma factors RpoS and FliA, activator protein LetE, and the LetA/S two-component system [98–100]. LetA/S signaling results in upregulation of two small non-coding RNAs, RsmY and RsmZ, which facilitate phase switching by repressing the global repressor CsrA [101–103].

Following differentiation into transmissive phase, *L. pneumophila* must egress the host cell. Mechanisms by which *L. pneumophila* temporally regulate egress are poorly understood. However, current data support a role for pore-formation and subsequent necrotic host cell death when *L. pneumophila* egress from macrophages and amoebae [104,105]. The Dot/Icm component IcmT contributes to pore-formation-dependent lysis of host cells; however, the mechanism by which IcmT functions is unknown[106]. The *Legionella* translocated effectors LepA and LepB also contribute to egress through non-lytic exocytosis from amoebae[107]. However, the mechanism by which Lep proteins facilitate release of *L. pneumophila* from protozoa is still unclear.

## Genome diversity and conserved proteins in *Legionella* virulence

### *Variation in* Legionella *genomes and effector repertoires*

A high degree of plasticity and diversity exists in the genomes of *Legionella* species. Recently, Gomez-Valero and colleagues sequenced the genomes of 58 *Legionella* species and performed a comparative analysis of genomes across 80 strains of *Legionella*[26]. *Legionella* genomes are highly diverse in size and content with genome size and GC content varying from 2.37 Mb to 4.88 Mb and 32.82% to 50.93%, respectively. The GC content of Legionella genomes is inversely correlated with genome size, suggesting that horizontal gene transfer, which results in AT-rich regions, drives *Legionella* genome size[26]. Moreover, out of 17,992 identified orthologous gene clusters, 5,832 (32%) were strain specific and only 1,008 genes (6%) comprised the core genome[26].

Despite diversity between *Legionella* genomes, the Dot/Icm T4SS is highly conserved and present in all species. However, the size and composition of *Legionella* effector repertoires is highly variable. For example, there is only a 50% overlap in effector repertoires of *L. pneumophila* and *L. longbeachae*[81]. No common set of effectors exists between strains that either cause human disease or replicate robustly in human macrophages[26]. Moreover, 18,000 unique translocated effectors are encoded by *Legionella* spp., which reflects the diversity of hosts and environmental adaptations evolved by *Legionella*[26].

Extensive co-evolution with diverse environmental phagotrophs has conferred on *Legionella* the ability to replicate within mammalian macrophages and cause human disease [7,35,108–110]. Using high-throughput genetic screening, Park and colleagues demonstrated that replication in *L. pneumophila* within mammalian cells is a consequence of combinatorial selection of virulence factors required for replication with phylogenetically diverse protozoan hosts[111]. The authors identified a subpopulation of effectors that are universally important for *L. pneumophila* replication within *A. castellanii, A. polyphaga, H. veriformis* and *N. gruberi*, and additional subpopulations important for replication within a single host genera or phylum. This study elegantly demonstrates how *L. pneumophila*’s broad host tropism has (1) driven evolution of the largest known repertoire of effector virulence factors discovered to date; and (2) how an environmental pathogen gained the ability to cause accidental human disease.

### *Eukaryotic-like domains and motifs in* Legionella *genomes*

*Legionella* species encode genes containing 137 distinct eukaryotic domains/motifs [26]. Ankyrin repeats are most prevalent eukaryotic motifs present across *Legionella* effectors; however, eukaryotic F-box, U-box, small GTPase, Rab, and SET domains are also abundant. The SET domain, present in histone methyltransferases, is present in 46 out of 58 species of *Legionella*. The effector RomA contains a SET domain and represses host gene expression via methylation of host histone proteins. Thus, the prevalence of SET domains among *Legionella* spp. suggests that host chromatin manipulation is a common mechanism employed by this genus [26,112].

Another motif prevalent in the genomes of multiple *Legionella* species is the ergosterol reductase ERG4/ERG24 motif. Ergosterol is present in the cell membranes of yeast, mitochondria, filamentous fungi and amoeba and 31 species encode one or two genes with the ERG4/ERG24 motif. Further sequence analysis revealed high similarity among *Legionella* proteins containing ERG domain with amoeba suggesting that *Legionella* has acquired this domain and others from amoebae[26]. However, the role of the ERG4/ERG24 motifs in *L. pneumophila* pathogenicity are poorly understood.

*Legionella* species encode 184 predicted small GTPases and 149 of these are present exclusively in eukaryotes and *Legionella*[26]. Homology was uncovered by BlastP analysis of *Legionella* Rab domain-containing proteins against protozoans in the NCBI database. Moreover, a subset of *Legionella* Rab GTPases possessed additional domains such as U-box, F-box and ankyrin repeats. Notably, 16 of these Rab GTPase motif-containing proteins were translocated by the Dot/Icm T4SS suggesting that these proteins are actual substrates of T4SS and function in the host cell[26]. While modulation of GTPase function is critical for intracellular replication, the functions of the majority of these genes is elusive. However, it is tempting to speculate a role for these proteins in subversion of host vesicular trafficking.

### Legionella *eukaryotic-like proteins*

In addition to proteins containing eukaryotic-like domains/motifs, *Legionella* additionally encode eukaryotic-like proteins [26,113]. Many of these proteins are confirmed or predicted Dot/Icm-translocated effectors. *Legionella* contains 2,196 eukaryotic-like proteins, representing 400 different orthologous groups with high similarity to eukaryotic proteins. The majority of these genes were likely acquired directly from protozoa, emphasizing the importance of host–pathogen interactions on *Legionella* genomes[26]. For example, *L. anisa* LanA0735 belongs to a FAD-dependent oxidoreductase family. This protein has a similarity to thioredoxin reductase, found in higher eukaryotes as two major isoenzymes: cytosolic and mitochondrial. In *Caenorhabditis elegans*, the cytosolic form has been reported to impede the lysosomal compartment acidification indicating a plausible role for LanA0735 in evasion of vacuole acidification during *Legionella* replication[114]. Moreover, *L. pneumophila* secretes eukaryote-like proteins PlcA and PlcB, which are phosphatidylcholine-hydrolyzing phospholipase C. Phosphatidylcholine is made by bacteria that interact closely with eukaryotes, such as *Brucella abortus* or *Francisella tularensis*. The synthesis of this phospholipid is essential for *L. pneumophila* virulence[115]. *Legionella* is unable to synthesize choline and these eukaryotic-like proteins likely aid in acquisition of choline from the host cell.

### *Conservation of the* Legionella *Dot/Icm T4SS and effectors*

Despite plasticity in the genomes of *Legionella* spp., the Dot/Icm T4SS is highly conserved in all *Legionella* spp. analyzed to date [25,26,109]. Between sequenced *Legionella* strains, Dot/Icm apparatus proteins share >50% amino acid identity with DotB and IcmS exceeding 90% identity[25]. Conversely, Dot/Icm-translocated effectors share little identity and the predicted number of effectors is highly variable between species. Only 8 core effectors are conserved in all analyzed *Legionella* genomes: Lpg0103 (VipF), Lpg1017 (RavC), Lpg0140, Lpg1356/Lpg1310, Lpg2300 (LegA3/AnkH/AnkW), Lpg2815 (IroT/MavN), Lpg2832 and Lpg3000 [25,26]. Seven additional effectors are present in all strains with the exception of a few strains, suggesting important role of these effectors in infection. Intraspecies effectors are highly conserved (82–97%), further emphasizing the influence of the host environment on evolution of the *Legionella* effector repertoire[25]. Together bacteria of the genus *Legionella* encode at least 18,000 effectors spanning 1,600 orthologous groups[26]. Thus, *Legionella* encode the greatest quantity and most diverse range of effectors among intracellular bacterial pathogens.

## Effector translocation by the Dot/Icm type IV secretion system

### Effector recognition by the Dot/Icm T4SS

The Dot/Icm T4SS is composed of 27 proteins, spans both bacterial membranes, and functions to translocate effector proteins directly into the host cell cytosol [116–120]. Effector translocation by the Dot/Icm T4SS involves recognition of effectors and subsequent translocation of unfolded effector proteins into host cells [121–124]. The Dot/Icm T4SS is composed of two major complexes: the core transmembrane complex (CTMC) and the Dot/Icm type IVB coupling complex (T4CC). The CTMC forms a pore for effector translocation and composed of DotC, DotD, DotF, DotG, DotH and DotK. The T4CC functions to recruit effectors for translocation and is composed of DotL (IcmO), DotM, DotN (IcmJ), IcmS, IcmW, LvgA, DotY, and DotZ. Six hetero-pentameric units (DotLMNYZ) of the T4CC form an inner membrane channel for the delivery of effectors [123,125–130]. Effector recognition by the T4CC is essential for translocation through the core transmembrane complex and cryo-electron tomography studies have revealed the molecular architecture of this structure and how effectors are recognized as translocation substrates [125,130].

Effector translocation through the T4CC occurs either through interaction with DotL-IcmSW or DotL-IcmSW-LvgA complexes or by DotM-mediated recognition of a C-terminal secretion signal, termed the E-block motif. DotL is a VirD4 homolog with an N-terminus ATPase domain and a C-terminal extension (CTE) that binds effectors in complex with IcmSW or IcmSW-LvgA [125,126,129,131]. The current model for IcmSW-mediated translocation is that effectors bound to IcmSW are delivered to the DotL channel where DotL ATPase activity may direct both effector unfolding and transport [125,126]. Some effectors are additionally recognized by LvgA, which binds IcmSW and copurifies together with the T4CC. Although many effectors are translocated independently of IcmSW or IcmSW-LvgA, *L. pneumophila* intracellular replication is severely attenuated by loss-of-function mutation in either *icmS, icmW* or *lvgA* [128,132]. DotM engages effectors that are translocated independently of IcmSW through recognition of a C-terminal Glu-rich region (E-block motif) [125,133]. While the C-terminal ~25 amino acids comprising the E-block motif are generally rich in Glu residues, residues with similar biochemical properties contribute more to translocation than Glu residues at specific positions[134]. Basic patches on DotM engage E-block-containing effectors through electrostatic interactions. This interaction is hypothesized to alleviate the requirement for IcmSW effector recognition [133]. Interestingly, the effector SidJ possesses an internal translocation signal, in addition to a C-terminal translocation signal, that aids in IcmSW-dependent Dot/Icm translocation[135]. Multiple signal sequences may provide an additional layer of effector translocation regulation. Thus, translocation signals are likely more complex than previously appreciated and may contribute to spatiotemporal regulation of effector translocation. How signal sequences and chaperone engagement contribute to translocation efficiency and hierarchy has yet to be elucidated.

### Spatiotemporal regulation of effector translocation

*L. pneumophila* effectors are translocated hierarchically during specific growth phases. Broadly, effectors are expressed either before/upon infection, early in infection, late in infection or during whole intracellular life cycle. Several effectors, including the SidE family, SidC and RalF, are accumulated during post-exponential phase, suggesting importance very early in infection [136–138]. In general, effector translocation hierarchy is thought to correlate with gene expression, but regulation of translocation is likely far more complex. Temporal regulation of effector translocation is important for *Legionella* intracellular replication and much remains to be discovered about how *Legionella* regulate translocation of effectors.

## Effector-mediated modulation of host autophagy, protein translation and ubiquitin homeostasis

### Regulation of host autophagy

Autophagy is an essential cellular process that is central to cellular survival and cell-autonomous defense against intracellular pathogens[139]. Several *L. pneumophila* effectors inhibit host autophagy, likely to subvert the lysosomal fusion with the LCV. RavZ disrupts autophagy thorugh irreversibly deconjugation of phosphatidylethanolamine from the autophagy-related ubiquitin like protein, LC3, which prevent LCV localization to autophagosomes[140]. Interestingly, RavZ-mediated LC3 delipidation is sufficient to impair autophagic targeting of other intracellular pathogens, including *Coxiella burnetii* and *Listeria monocytogenes* [141,142]. Autophagy is also impaired through the action of a *L. pneumophila* effectors S1P-lyase (LpSPL; LegS2) and Lpg1137, via modulation of sphingolipid metabolism and cleavage of the autophagy-associated SNARE syntaxin-17, respectively [143,144]. Paradoxically, the *L. pneumophila* effector LegA9, an ankyrin-containing protein, upregulates autophagy and contributes to macrophage restriction of *L. pneumophila* through an unknown mechanism[145]. Thus, autophagy is central to *L. pneumophila* intracellular replication and further investigation will likely reveal additional sophisticated mechanisms employed by *L. pneumophila* to subvert host autophagy.

### Inhibition of host protein synthesis

Seven *L. pneumophila* effectors, Lgt1-3, SidI, SidL, LegK4 and RavX, are capable of inhibiting eukaryotic protein synthesis (reviewed in [146]). Lgt1-3 are glycosyltransferases that modify eukaryotic GTPase elongation factor 1A (eEF1A), an essential component of the eukaryotic protein translation elongation complex. Lgt1-3 glycosylate eEF1A at Ser-53 through covalent addition of a glucose moiety. Ser-53 is conserved in eukaryotes and located in the GTPase domain of eEF1A. Ectopic expression of Lgt1 in yeast is cytotoxic due to inactivation of eEF1A [147–150]. Moreover, Lgt1-3 cooperate with the SidE family of effectors to facilitate acquisition of essential host-derived amino acids (see below)[151].

The effector SidI is a predicted glycosyltransferase capable of hydrolyzing GDP-mannose. SidI interacts with eEF1A and eEF1Bγ. However, interaction with eEF1A is not sufficient for SidI-mediated translation inhibition. eEF1A additionally upregulates the eukaryotic heat shock response through interaction with heat shock regulatory protein 1 (HSF1) [152], and SidI is sufficient for induction of the host stress response[153]. However, translation inhibition alone is insufficient to induce the heat shock response[153]. Since SidI-mediated protein translation inhibition is suppressed by its metaeffector, MesI, translation inhibition may not be the bona fide function of SidI within host cells[154]. Although SidI is not individually required for *L. pneumophila*, its enzymatic activity is uniquely deleterious to *L. pneumophila* intracellular replication in the absence of MesI [153,155]. The mechanism by which SidI prevents L. pneumophila intracellular replication is currently unknown.

The effector kinase, LegK4 impairs host translation through phosphorylation of cytosolic Hsp70. Phosphorylated Hsp70 associates with translating polysomes but has attenuated ATPase activity, which impairs its refolding capacity and, consequently, protein translation[156]. Detailed molecular mechanisms by which SidI, SidL and RavX inhibit host protein translation are currently unknown.

Inhibition of host translation is hypothesized to facilitate acquisition of essential nutrients from host cells[151]. However, inhibition of protein translation also contributes to restriction of *L. pneumophila* via effector-triggered immunity infection models (see below) [157–160]. Thus, subversion of host translation is central to *L. pneumophila* pathogenicity but also facilitates pathogen detection and restriction by macrophages.

### Modulation of host ubiquitination pathways

*L. pneumophila* intracellular replication hinges on effector-mediated modulation of cellular ubiquitin pathways. Subversion of ubiquitin pathways involves the concerted action of several effectors, most of which are indispensable for intracellular replication. Comprehensive and detailed reviews are available on subversion of ubiquitination pathways by *L. pneumophila* effectors [161,162]. Ubiquitination is regulated by *L. pneumophila* effectors through novel enzymatic activity and molecular mimicry of eukaryotic ubiquitin ligases and deubiquitinases [163–170]. Of note is the recent discovery of novel effector-mediated ubiquitin modulation mechanisms. The SidE family of effectors (SidE/SdeABC) and their metaeffector, SidJ, cooperate to facilitate LCV biogenesis. The SidE family of effectors are mono-ADP-ribosyltransferases that ligate ubiquitin to ER associated Rab GTPases independently of host E1 and E2 enzymes [166,171]. The SidE family ubiquitinate ER-associated Rab GTPases, Rag GTPases and host reticulon 4 to regulate tubular ER dynamics for biogenesis of the LCV and activity of the mechanistic target of rapamycin complex 1 (mTORC1; see below) [151,166,171]. SidJ utilizes host calmodulin as a co-factor to polyglutamylate and inactivate SidE effectors [165,172,173]. SidJ-mediated regulation of the SidE family is critical for intracellular replication in natural and accidental hosts [174,175].

The effector deamidases MavC and MvcA are functional antagonists that temporally regulate ubiquitination and activity of the host E2 enzyme Ube2N. MavC catalyzes E1-independent monoubiquitylation and inhibition of Ube2N [176]. However, Ube2N activity is restored through the action of MvcA, which deubiquitinates Ube2N-Ub [177]. In mammalian cells, Ube2N inactivation impairs ubiquitination and proteasomal degradation of the host inhibitor of κB03B1 (IκBα), which restricts NF-κB-mediated gene expression (see below)[167]. MavC and MvcA are both regulated by a metaeffector, Lpg2149, which binds and inhibits the deamidase activity of both effectors [176]. Increased expression of *mavC* in transmissive phase bacteria overcomes Lpg2149-mediated restriction and facilitates temporal regulation of Ube2N activity [176]. MavC and MvcA are individually dispensable for *L. pneumophila* intracellular replication, which is likely a consequence of functional redundancy with other effectors in the host systems tested.

## Effector-mediated nutrient acquisition and modulation of host metabolism

*L. pneumophila* is reliant on host-derived amino acids for intracellular replication [178–180]. *L. pneumophila* is auxotrophic for valine, threonine, serine, leucine, methionine, arginine, isoleucine and cysteine, which is a preferred source of carbon during intracellular replication [181,182]. The effector AnkB facilitates acquisition of host-derived amino acids through recruitment of host polyubiquitinated (polyUb) proteins to the surface of the LCV. AnkB contains a CaaX motif, which is farnesylated and anchors the effector to the LCV surface, ankyrin repeats and an F-box domain. The ankyrin and F-box domains facilitate attachment of host polyUb proteins to the LCV membrane, which are subsequently proteolyzed by host 26S proteasome [170,183]. Both genetic deletion of *ankB* and chemical inhibition of 26S proteasomes impair *L. pneumophila* intracellular replication and supplementation of cell culture with free amino acids rescues these growth defects[184]. Thus, effector-mediated subversion of the ubiquitin-proteasome system facilitates *L. pneumophila* nutrient acquisition from host cells[186].

**Figure 2.**
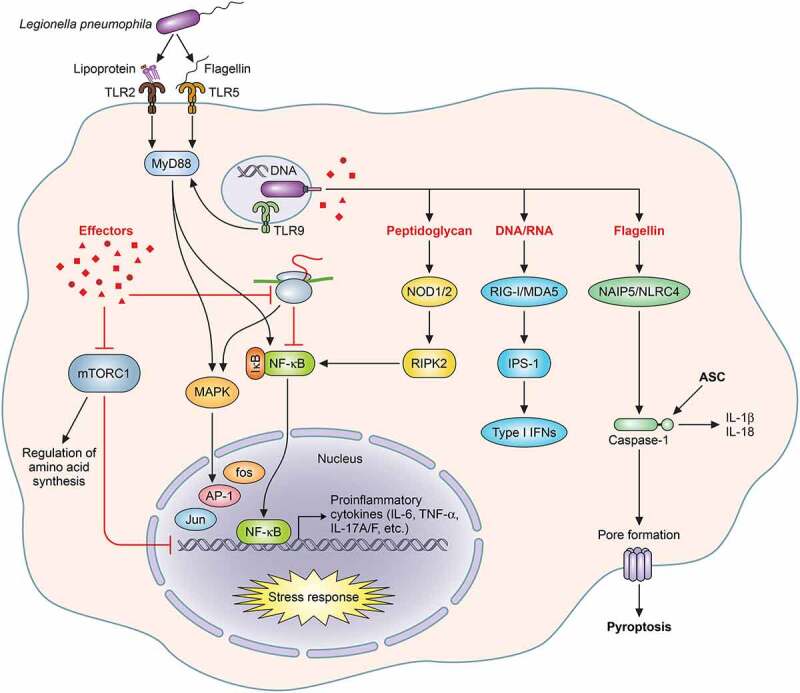
**Innate immune signaling initiated by *L. pneumophila* within macrophages**. The schematic represents the activation of multiple pathways upon mammalian phagocyte infection with *L. pneumophila. Legionella*-associated molecular patterns are recognized via pattern recognition receptors (PRRs) of the phagocyte. Activation of PRRs and cytosolic sensors triggers downstream molecules and processes that eventually lead to restriction of *L. pneumophila* replication. Specifically, TLR2, TLR5 and TLR9 discern bacterial lipoprotein, flagellin and dsDNA, respectively, which activate downstream NF-kB mediated proinflammatory cytokine response. Effector-dependent translation inhibition activates NF-κB and MAPK signaling to initiates a proinflammatory transcriptional response. *Legionella* effectors also inhibit the mTORC1 complex which negatively affects the amino acid synthesis and proinflammatory cytokine production. *Legionella* flagellin is recognized by the NAIP5/NLRC4 inflammasome and downstream activation of caspase-1 leads to pyroptosis and IL-1β/IL-18 cytokine release. NOD1/2 recognizes degradative products of bacterial peptidoglycan, eliciting RIPK2-dependent NF-κB activation and proinflammatory cytokine production. Bacterial DNA/RNA is sensed in the host cell cytosol by RIG-I and MDA5 which leads to type I interferon production

*L. pneumophila* relies on host-derived amino acids, but free amino acid levels are tightly regulated in eukaryotic cells. mTORC1 is a conserved complex composed of mTOR kinase and several regulatory enzymes that is regulated in part by availability of amino acids and other nutrients (Figure 2)[185]. Upon activation, mTORC1 controls several cellular processes including repression of autophagy, translation initiation and lysosome biosynthesis[186]. The Lgt and SidE effector families (see above) act antagonistically toward mTORC1. Lgts-mediated translation inhibition results in mTORC1 activation whereas the SidE family of effectors negatively regulate mTORC1 by ubiquitination and inhibition Rag GTPases, which contribute to amino-acid sensing by mTORC1 and liberation of host amino acids for bacterial intake[151].

Intracellular *L. pneumophila* acquire iron from the host cell via the function of the effector MavN/IroT. MavN/IroT spans the LCV membrane and functions to transport iron into the LCV [189]. *L. pneumophila* strains lacking *mavN/iroT* are attenuated for intracellular growth and exhibit characteristics of iron starvation [187,188]. MavN/IroT is one of very few *L. pneumophila* effectors universally required for intracellular replication and its activity provides insight into mechanisms of iron acquisition by intravacuolar pathogens[189].

*L. pneumophila* effectors also directly modulate host cell metabolism. The effector LamA subverts glucose metabolism to regulate encystation of amoebae, which occurs as a result of environmental stress. Amoebal cysts are hypothesized to enhance environmental stability of intracellular *L. pneumophila*. However, although retained in a viable state, *L. pneumophila* is unable to replicate within amoebal cysts [189–191]. Price *et al*. recently reported that LamA, an effector amylase, induces a “hyper-glucose” state in host cells via degradation of host glycogen. Consequently, amoebae are unable to synthesize the cellulose-rich cyst wall[192]. In human macrophages (hMDMs), LamA triggers a pro-inflammatory response that moderately restricts bacterial replication. LamA mediated high glucose levels in hMDMs shift the metabolism to aerobic glycolysis which directly triggers a rapid M1-like pro-inflammatory polarization and pro-inflammatory cytokine production. Moreover, LamA augments IFN-γ-mediated IDO1 activity, which depletes cellular tryptophan. Although *L. pneumophila* is not auxotrophic for tryptophan, host-derived tryptophan is important for *L. pneumophila* replication within macrophages [192,193].

A recent study also revealed that *L. pneumophila* encodes an effector ADP-ribosyltransferase that modifies a class of host NAD+-dependent glutamate dehydrogenases (GDH). The effector *Legionella* ADP-ribosyltransferase 1 (Lart1; Lpg0181) ADP-ribosylates GDH on a conserved arginine within the nucleotide-binding pocket, which renders GDH inactive[194]. However, the role of Lart1-mediated ADP-ribosylation of GDH during *L. pneumophila* infection has not been fully elucidated. A *L. pneumophila* ∆*lart1* mutant is not impaired for replication within *A. castellanii*, suggesting that within this host, Lart1 functions redundantly with other effectors. This study has uncovered an additional mechanism by which *L. pneumophila* may subvert host cell metabolism.

Thus, *L. pneumophila* has evolved an extensive repertoire of effectors that regulate diverse host cell processes. This work has collectively shed light on mechanism of *Legionella* virulence and broad themes in host–pathogen interactions.

## The *Legionella* type II secretion system

*L. pneumophila* encodes a type II secretion system (T2SS), also called the *Legionella* secretion pathway (Lsp), which is important for virulence and persistence in the environment [195]. T2SSs are highly conserved, evolutionarily related to bacterial type IV pili, and broadly distributed among members of the phylum [196–199]. The T2SS plays a crucial role in bacterial pathogenicity by exporting various virulence factors, toxins, lipases, proteases, chitinases and novel proteins outside the bacterial cell [198]. Prior to secretion via T2SS, unfolded or folded protein substrates enter the periplasm through either the Sec translocon or the twin-arginine translocon (Tat), respectively. The T2SS machinery is comprised broadly of four subcomplexes composed of 12 core proteins: T2S C, D, E, F, G, H, I, J, K, L, M and O [200,201]. The first subcomplex is a “secretin,” which facilitates substrate translocation across the outer membrane and is composed of T2S D oligomers. These T2S D oligomers are associated with the second subcomplex, an inner membrane heterooligomer comprised of T2S F, L, and M multimers, which creates a periplasmic channel. The inner and outer membrane complexes are coupled by a “clamp protein,” T2S C. The third subcomplex, a periplasmic “pseudopilus,” consists of T2G G, H, I, J and K components, which may function as a “piston” to propel the substrates through the outer membrane subcomplex. The fourth subcomplex is an ATPase and hexamer of T2S E protein. Finally, T2S O, an inner membrane prepilin peptidase allows pseudopilin maturation [201,202].

The *Legionella* T2SS is important for both intra and extracellular survival of *L. pneumophila*. It plays a crucial role in biofilm formation, intracellular replication in amoeba and macrophages, suppression of cytokine response from infected cells, growth and persistence in the murine lungs [195,199,203–208]. Over 25 T2SS substrates have been identified in *L. pneumophila* [199,202]. These substrates include ProA, PlaA, Map, PlaC (acetyltransferase), PlcA (phospholipase C), SrnA (ribonuclease), ChiA (chitinase), CelA (cellulase), LapA, LapB (aminopeptidases), and NttA,B,C,D,E,G [204,209–212]. NttA,C,D,E are required for *L. pneumophila* replication within multiple species of amoebae[212]. The T2SS substrates LegP and Map contain eukaryotic-like protease motifs [204,213]. Interestingly, LegP is a confirmed substrate of the Dot/Icm T4SS [68], but the mechanism by which a single protein could serve as a substrate for both secretions systems is unknown. The T2SS substrates ProA, PlaC and SrnA are necessary for optimal infection in *H. vermiformis* and *N. lovaniensis* whereas NttA is required to replicate in *A. castallanii* [209,210,214]. These observations suggest that T2SS substrates may shape the host range of *L. pneumophila*.

The T2SS contributes to *L. pneumophila* virulence in cultured mammalian cells and mouse models of LD [203,215]. Loss-of-function mutation of the T2SS genes *lspF, lspDE, lspG, lspK*, and *lspO* impairs *L. pneumophila* infection of human macrophage cell lines, mouse macrophage cell lines, primary BMDMs and alveolar epithelial cells [203,205,215–218]. The T2SS substrate, ChiA, is important for survival in the mouse lung [204,209]. ChiA is a chitinase that possesses additional peptidase activity and degrades mucin *in vitro* [208]. Degradation of mucin *in vivo* may enhance *L. pneumophila* motility in airways and enhance access to alveolar macrophages. Another substrate, ProA, causes lung tissue damage and transferring degradation that can enhance iron acquisition [204,219–221]. The contribution of the full range of T2S substrates to *L. pneumophila* virulence has yet to be elucidated, but current data support an essential role for this secretion system and its substrates in infection.

The T2SS additionally attenuates the mammalian innate immune response. T2SS function decreases cytokine and chemokine levels in the supernatants of macrophages and epithelial cells and within the mouse lung during *L. pneumophila* infection [205,215]. The T2SS decreases cytokine abundance via transcriptional and post-transcriptional mechanisms. The metalloprotease ProA dampens cytokine production at the post-transcriptional level; however, substrates responsible for attenuation of gene expression have yet to be identified[215]. Moreover, a role for the T2SS in TNF-α -mediated macrophage defense against *L. pneumophila* was demonstrated by restoration of T2SS mutant growth upon antibody neutralization of TNF[215]. This suggests that T2SS promotes *L. pneumophila* growth in macrophages and epithelia by dampening cytokine response in addition to some unidentified mechanisms. The evolutionary basis T2SS-mediated attenuation of inflammation is unclear since amoebae lack pro-inflammatory genes and signaling cascades. Whether the attenuated inflammatory response is due to serendipitous function of T2SS substrates or macrophage response to infection is unclear.

Truchan *et al.* recently observed localization of the T2SS substrates ProA and ChiA to the cytoplasmic face of the LCV membrane (LCVM)[222]. The authors hypothesize that translocation of ProA and ChiA to the LCVM is results from LCV permeability, as observed by galectin-3 accumulation around the LCV. Interestingly, this phenomenon was observed in human but not mouse macrophages. ProA and ChiA localized to the LCVM in U937 cells and differentiated polymorphonuclear cells (PBMCs) but not in BMDMs from permissive mouse strains. Mechanisms by which the LCV becomes semipermeable and the observed differences between mouse and human macrophages are unclear.

Taken together, T2SS contributes to *L. pneumophila* virulence via environmental survival and persistence in the host. Future studies are required to completely understand the role of T2SS substrates in disease progression and to define the underlying mechanisms of action.

## Host immune responses to *Legionella* infection

### *Macrophage detection of* L. pneumophila *infection*

*L. pneumophila* activates an orchestrated and robust inflammatory response during infection of healthy hosts. Inbred mouse models of *L. pneumophila* infection and LD have been pivotal defining mechanisms of host defense against intracellular pathogens. *L. pneumophila* has therefore emerged as an invaluable model pathogen to study host defense mechanism including innate immunity, inflammasome activation and acute lung inflammation.

Innate immune detection of *L. pneumophila* involves synergistic recognition of pathogen-associated molecular patterns (PAMPs) by pattern recognition receptors (PRRs) including toll-like receptors (TLRs), nucleotide-binding oligomerization domain-like receptor (NLRs), and Rig-like helicases (RLH) that results in the activation of immune responses and pathogen clearance (Figure 2) [223,224]. Pro-inflammatory gene expression through NF-κB and AP-1 (Jun/Fos) downstream of TLRs is mediated through signal cascades involving the adaptors MyD88/TRIF and mitogen activated kinases (MAPKs). MyD88 triggers a signaling cascade that results in upregulation of pro-inflammatory gene expression [225,226]. MyD88-deficient mice are highly susceptible to *L. pneumophila* infection and due to severe defects in leukocyte recruitment, proinflammatory cytokine and chemokine secretion [227–230]. Intracellular pathogen detection is also facilitated through inflammasome activation and effector-mediated immunity. Detection of *L. pneumophila* results in upregulation of a complex and highly orchestrated inflammatory response *in vivo* that involves both myeloid and somatic cells.

#### *Role of PAMP recognition in* L. pneumophila *replication*

TLR2, TLR4, TLR5 and TLR9 contribute to innate immunity against *L. pneumophila* through activation of signal transduction cascades culminating in NF-κB and AP-1-mediated pro-inflammatory gene expression [228,230–233] (Figure 2). Interestingly, TLR4, which recognizes lipopolysaccharide (LPS), does not play a major role in *L. pneumophila* recognition [231,234,235]. TLR2-deficient mice have delayed production of proinflammatory cytokines and neutrophil recruitment during *L. pneumophila* infection[228]. TLR5 detects bacterial flagellin and enhances neutrophil recruitment to the *L. pneumophila*-infected lung at early time points post-infection [230,232]. TLR9 signaling additionally contributes to *L. pneumophila* but likely functions redundantly with TLR5. Interestingly, TLR9 signaling is more important for restriction of *L. pneumophila* in Balb/c mice than in A/J mice, likely owing to differences in the genetic backgrounds of these hosts [230,236]. TLR signaling synergizes with intracellular PAMP detection for optimal induction of inflammation and restriction of *L. pneumophila* intracellular replication.

Nucleotide-binding and oligomerization domain protein 1 (NOD1) and NOD2 consist of a N-terminal interaction domain, a nucleotide binding central domain and a leucine-rich repeat (LRR) C-terminal variable domain[235]. NOD1 and NOD2 detect cytosolic peptidoglycan and initiate signaling through activation of receptor interacting protein kinase 2 (RIP2), which culminates in activation of NF-κB (Figure 2) [226,235]. Loss of RIP2-mediated signaling impairs Dot/Icm-dependent immune responses but has only modest effects on pro-inflammatory cytokine production by BMDMs[237]. Loss of RIP2-mediated signaling enhances *L. pneumophila* bacterial burden in the mouse lung, likely through impaired neutrophil recruitment[238].

Retinoic-acid inducible gene-I (RIG-I)-like helicases (RLHs) melanoma-differentiation-associated gene-5 (MDA5) and RIG-I also contribute to macrophage detection of *L. pneumophila* through detection of cytosolic nucleic acids. RIG-I/MDA5 activation results in signal transduction involving multiple adaptor proteins, including interferon-β promoter stimulator-1 (IPS-1) (Figure 2) [235,239]. This in turn activates NF-κB and interferon regulatory factors (IRFs), which enhance the production of proinflammatory cytokines and type-I interferons (IFN-I), respectively [235,241]. *L. pneumophila* activates this pathway via RNA translocation into the host cell[240]. Interestingly, despite increased IFN-I during *L. pneumophila* infection, impaired IFN-I receptor (IFNAR) signaling does not affect *L. pneumophila* replication in the lungs [240,241].

#### *Role of inflammasomes in* L. pneumophila *infection*

Inflammasomes are large intracellular protein complexes that recognize pathogens and cellular stressors. Generally, pathogen sensing by inflammasomes results in activation of effector caspases, caspase-1 and caspase-8, the consequence of which is pyroptosis and secretion of IL-1β and IL-18[242]. The NAIP5/NLRC4 inflammasome detects bacterial flagellin and is responsible for potent restriction of *L. pneumophila* by wild-type C57BL/6 macrophages (Figure 2) [243,244]. *L. pneumophila* has been instrumental in delineating structural and functional insights into flagellin detection by NAIP5/NLRC4 and consequent pathogen restriction [245–247]. A comprehensive review detailing mechanisms of inflammasome activation by *L. pneumophila* is available[247]. However, a critical role for pyroptosis in host restriction *of L. pneumophila* was recently described. Pyroptosis occurs through caspase-1 cleavage of Gasdermin D (GSDMD). Cleaved GSDMD forms pores in the plasma membrane and culminates in release of cytosolic components, cytokines and eventual cell lysis[248]. NLRC4-mediated restriction of *L. pneumophila* is facilitated through activation of caspase-7 and GSDMD-mediated pyroptosis [249,250]. The mechanism by which caspase-7 contributes to restriction of *L. pneumophila* has yet to be fully elucidated, but its role in cell death further suggests an essential role for inflammatory host cell death in NAIP5/NLRC4-mediated restriction of *L. pneumophila*.

### *Cytokine responses during* L. pneumophila *infection and bystander responses*

Pro-inflammatory cytokines are pivotal for host defense against *L. pneumophila*. Of these cytokines, IFN-γ and TNFα are primarily responsible for immune clearance of *L. pneumophila*. High concentrations of IFN-γ are generated in the mouse lung in response to *L. pneumophila* infection, the majority of which is made by natural killer (NK) cells in a MyD88-dependent manner[229]. NK cells make IFN-γ in response to IL-12 and IL-18; however, loss of IL-18 only has a modest effect on IFN-γ production and bacterial burden in the lung [230,251]. Conversely, monocyte generated IL-12 is crucial for IFN-γ production by NK cells in the *Legionella*-infected lung [230,252]. IFN-γ-deficient mice are unable to clear pulmonary *L. pneumophila* and, in contrast to wild-type strains, will succumb to infection [230,253]. IFN-γ facilitates cell-autonomous restriction of *L. pneumophila* through upregulation of interferon-stimulated genes, oxidative stress, nutritional remodeling and xenophagy[254]. Interferon-stimulated immune responsive gene 1 (IRG1) additionally restricts *L. pneumophila* through the production of itaconic acid[255]. Thus, IFN-γ production by non-infected cells drives cell-autonomous macrophage restriction of *L. pneumophila*.

In addition to IFN-γ, high levels of TNFα are generated in response to *L. pneumophila* infection in the lung[256]. Loss of TNF-mediated signaling impairs pulmonary bacterial clearance and survival of *L. pneumophila*-infected mice [256,257]. In the lung, TNF is produced primarily by uninfected inflammatory monocytes and neutrophils but activate antimicrobial defenses in infected macrophages[252]. TNFα signals through TNFR1 and TNFR2; however, TNFR1-mediated signaling plays a more central role in host defense against *L. pneumophila*[258]. Signaling through TNFR1 enhances macrophage antimicrobial activity through enhanced lysosomal fusion with LCVs. TNFα synergizes with IFN-γ and IFN-I to restrict *L. pneumophila*[258].

Several *L. pneumophila* effectors (Lgt1-3, SidI, SidL, LegK4 and RavX; see above) inhibit host protein synthesis, which impairs cytokine production during infection. However, a robust pro-inflammatory response in generated. In the *L. pneumophila*-infected lung, infected and uninfected cells produce different sets of cytokines. Despite potent effector-mediated translation inhibition, *L. pneumophila*-infected cells secrete IL-1 through mRNA superinduction[160]. IL-1 receptor (IL-1 R)-mediated signaling is required for the production of proinflammatory cytokines (IL-12, TNFα, and IL-6) by bystander monocytes and neutrophils[159]. Recent work from Shin and colleagues revealed how IL-1 promotes bystander pro-inflammatory cytokine production. IL-1 R signaling stimulates the production of granulocyte colony-stimulating factor (GM-CSF) by the alveolar epithelium. GM-CSF signaling synergizes with TLR-mediated signaling to enhance aerobic glycolysis, which enhances pro-inflammatory cytokine production by bystander cells and consequent activation of infected alveolar macrophages[259]. Thus, the inflammatory response to *L. pneumophila* in vivo is highly orchestrated and involves myeloid and somatic cells.

Interestingly, the *L. pneumophila* effector LegC4, which is important for replication within *A. castellanii*, paradoxically attenuates *L. pneumophila* fitness in the mouse lung. This is likely through exacerbation of cytokine-mediated restriction in macrophages, but the mechanism by which this occurs is unknown [155,260].

### *The adaptive immune response to* L. pneumophila

Adaptive immunity additionally contributes to host defense against *L. pneumophila*. CD4+ and CD8 + T cells contribute to host defense against *L. pneumophila*[261]. Following mediastinal lymph node priming with *L. pneumophila*, differentiated Th17 and Th1 T cells infiltrate lungs and produce IL-17 and IFN-γ, respectively [262]. Furthermore, Th17-mediated restriction of *L. pneumophila* depends on NLRC4 inflammasome and MyD88, whereas the Th1 response is initiated in the absence of MyD88[262]. CD8 + T cells are additionally a source of IFN-γ and promote M1 macrophage skewing[263].

Mice mount a humoral response to *L. pneumophila* introduced either via intranasal inoculation or intravenous injection[264]. After primary exposure to *L. pneumophila*, mice generate IgA and IgG responses in the bronchoalveolar lavage fluid and serum, respectively, followed by the establishment of memory B cells in the lung[264]. Thirty novel *L. pneumophila*-specific B cell antigens have been identified and include components of bacterial membrane[265]. Immunization of guinea pigs with *L. pneumophila* Hsp60 or OmpS results in partial protection from *L. pneumophila* infection [265,266]. However, the adaptive immune response to *Legionella* infection is considerably feeble and does not provide prolonged immunity against the pathogen since reinfection has been reported [267]. Altogether, these studies demonstrate the importance of innate immunity in host restriction of *L. pneumophila* and elaborate challenges associated with the development of lasting immunity in susceptible individuals.

## Concluding remarks and future directions

Past four decades of research on *Legionella* have brought important insights into the virulence strategies and mechanisms employed by *Legionella* to replicate within environmental and mammalian phagocytes and cause disease in humans. To date, the genus *Legionella* has the largest and most diverse effector repertoire amongst intracellular pathogens. Despite the diversity among *Legionella* species, there are common themes in their virulence strategies including Dot/Icm effector translocation and acquisition of eukaryotic-like effectors that have resulted from extensive co-evolution with protozoa. *Legionella* has also emerged as an invaluable model pathogen to understand innate immunity and major breakthroughs have been made using mouse models of LD. Despite these major advances, many questions remain open. The study of *Legionella* bacteria is intriguing in many ways and future studies will teach us not only about bacterial pathogenicity but also eukaryotic cell biology and mammalian antimicrobial immune defenses. We are excited for what the next four decades will reveal about *Legionella* virulence.

## Abbreviations

*Legionella*; Legionnaires’ Disease; pathogenicity; virulence; Dot/Icm secretion system; effector proteins; host–pathogen interactions; host response; innate immunity; effector-mediated immunity
